# Attitudes of stakeholders in psychiatry towards the inclusion of children in genomic research

**DOI:** 10.1186/s40246-018-0144-8

**Published:** 2018-03-05

**Authors:** Anna Sundby, Merete Watt Boolsen, Kristoffer Sølvsten Burgdorf, Henrik Ullum, Thomas Folkmann Hansen, Ole Mors

**Affiliations:** 1 Department of Clinical Medicine, Psychosis Research Unit, Aarhus University Hospital, Skovagervej 2, 8240 Risskov, Denmark; 2 The Lundbeck Foundation Initiative for Integrative Psychiatric Research, iPSYCH, Copenhagen, Denmark; 3 Department of Political Science, Copenhagen University, Copenhagen, Denmark; 4 Department of Clinical Immunology, Copenhagen University Hospital, Copenhagen, Denmark; 5 Institute for Biological Psychiatry, Mental Health Centre Sct. Hans, Copenhagen University Hospital, Copenhagen, Denmark; 6 Danish Headache Center, Department of Neurology, Rigshospitalet-Glostrup, Copenhagen, Denmark

**Keywords:** Child, Minors, Attitude, Whole genome sequencing, Ethics research, Mental disorders

## Abstract

**Background:**

Genomic sequencing of children in research raises complex ethical issues. This study aims to gain more knowledge on the attitudes towards the inclusion of children as research subjects in genomic research and towards the disclosure of pertinent and incidental findings to the parents and the child.

**Methods:**

Qualitative data were collected from interviews with a wide range of informants: experts engaged in genomic research, clinical geneticists, persons with mental disorders, relatives, and blood donors. Quantitative data were collected from a cross-sectional web-based survey among 1227 parents and 1406 non-parents who were potential stakeholders in psychiatric genomic research.

**Results:**

Participants generally expressed positive views on children’s participation in genomic research. The informants in the qualitative interviews highlighted the age of the child as a critical aspect when disclosing genetic information. Other important aspects were the child’s right to an autonomous choice, the emotional burden of knowing imposed on both the child and the parents, and the possibility of receiving beneficial clinical information regarding the future health of the child. Nevertheless, there was no consensus whether the parent or the child should receive the findings. A majority of survey stakeholders agreed that children should be able to participate in genomic research. The majority agreed that both pertinent and incidental findings should be returned to the parents and to the child when of legal age. Having children does not affect the stakeholder’s attitudes towards the inclusion of children as research subjects in genomic research.

**Conclusion:**

Our findings illustrate that both the child’s right to autonomy and the parents’ interest to be informed are important factors that are found valuable by the participants. In future guidelines governing children as subjects in genomic research, it would thus be essential to incorporate the child’s right to an open future, including the right to receive information on adult-onset genetic disorders.

## Background

Genome sequencing, i.e., whole genome and whole exome sequencing, has now become a widely used tool in research, for example, in psychiatric genetic research. However, genome sequencing generates huge amounts of genomic information, and some of the information is clinically useful. There is an ongoing debate regarding the return of individual genetic research findings unrelated to the condition under investigation [1–7]. The community of clinical genetics refers to these types of results as incidental findings [8]. Because incidental findings should be expected as a part of genomic sequencing, other terms as secondary findings, additional findings, and non-pertinent findings have been suggested [8, 9]. However, the increasing number of large-scale genomic research projects and the inclusion of children in genomic research have made it important to continue the debate regarding the return of individual genetic research findings.

Children are included in genomic research because some disorders are childhood-onset [10]. For example, to study genetic factors for attention deficit hyperactivity disorder (ADHD) or autism spectrum disorder, blood and buccal cell samples from children with ADHD and autism spectrum disorder are often taken in the clinical setting and used for research. Research on children can also be done using already existing biological samples stored in a biobank. For example, the Danish Neonatal Screening Biobank holds archived blood samples from all newborns in Denmark after screening for phenylketonuria (PKU) and several other congenital diseases [11, 12]. The dried blood spot samples from the neonates provide an opportunity to use DNA from children in research on a very large scale [13].

The ethical issues of including children in genomic research differ from those raised when including adults [10, 14, 15]. One of the biggest differences is that a third party (the parent) must mediate the relationship between the researcher and the research subject (the child) [10, 14, 16, 17]. Furthermore, young children do not have the same capacity to understand the research information or the implications of the findings. Common questions related to involvement of children in genomic research include the following: Should children be research subjects at all? Should the findings be returned to the child or to the parents? [5, 10, 14, 17–22].

If samples are to be collected and used for research purposes in Denmark, the research subject must be of legal age (18 years) to voluntarily consent to participate. If the research subject is not able to consent, for example, due to the age, such authority lies with the parents or the guardians [23]. If a research project obtains significant information about the health status of the research subject, the research subject should be informed, unless the subject has specifically expressed that s/he does not want the information [24]. The Danish National Committee on Health Research Ethics has developed a guideline on genomic research [25]. The guideline addresses inclusion of children in genomic research and return of incidental findings. It is emphasized that there must be a direct health benefit for the individual child to justify inclusion in whole genome research and that it is not sufficient to include children simply to study the genetics of a disease. Return of results to research subjects is only the norm for genetic variants with high penetrance that predispose to serious curable or treatable disease, whereas no return of results is usually provided for genetic variants with low or moderate penetrance and uncertain clinical significance [25]. The guideline furthermore addresses that exemptions from obtaining consent from parents can be given if the research project uses biological samples archived in a biobank [25].

The legal regulations in Denmark on the return of findings based on children’s DNA are complex and unclear. This makes it difficult for researchers and research subjects to navigate the regulations governing the return of findings from children’s DNA. We assume that the severity, treatability, and preventability of the disease; the age of the child; and the decision on whether to return incidental findings are discussed at the time of donation of the sample as all these issues are relevant in the decision-making. It is unclear whether the return of genetic risk variants with high penetrance that predispose to severe, curable, or treatable diseases encompasses all known genetic risk variants, including those for adult-onset disorders. Furthermore, it is unclear if results should be returned to the child or to the parents or whether results should be conveyed only when the child reaches legal age.

Previous studies have found that parents generally think that children should be involved in genomic research and that parents have a right to know about incidental findings concerning their child(ren) [26–28]. However, to our knowledge, no large studies have investigated the attitudes towards the involvement of children in genomic research among potential research subjects. Researching such attitudes is important to create policy strategies and to design future genomic research projects that meet the concerns and expectations of potential research subjects. We chose to study this topic in the context of mental disorders because they are some of the commonest disorders in the population and because large genome sequencing studies have been conducted in this area [13, 29, 30].

This study aims to explore attitudes among stakeholders in psychiatric genetic research towards (i) children as genomic research subjects, (ii) return of pertinent and incidental findings, and (iii) disclosure to participants (i.e., children) or their parents.

## Methods

For the purpose of this study, we use pertinent findings for the results that are directly relevant to the condition under investigation and incidental findings for results that are not directly related to the research project but may have health importance for the individual research subject. We use a definition of childhood, starting from birth to 18 years of age, referring to the legal age of majority in Denmark. According to Statistics Denmark, individuals under age 26 living at home are defined as home-living children because the child has parental reference to at least one adult [31]. Therefore, we define a parent as a male or female with children under age 26 years living at home.

### Mixed-methods

We used a mixed-methods design as we collected both qualitative and quantitative data [32]. This approach was taken to address the overall aim at different levels and to complement the strengths of a single-method design. The motivation for using a mixed-methods design was to use qualitative interviews to develop and modify the quantitative survey, but the qualitative interviews also helped to approach the field regarding inclusion of children in genomic research from a different angle.

## The qualitative interviews

The interviews were conducted to explore the attitudes towards ethical issues regarding the use of genomic sequencing in psychiatric research among experts engaged in genomic research and among potential research participants in psychiatric genetic research. The interview guides were developed on the basis of relevant literature and the research questions of the overall study. The overall interview guides focused on attitudes towards (i) sharing findings, (ii) consenting procedures, (iii) duties of genomic researchers, (iv) including children in genomic research, and (v) misuse of knowledge in genetics. All interviews were conducted from 5 December 2012 to 11 December 2013, and each interview lasted between 30 and 90 min.

### The informants

The informants in the interviews were experts (doctors and nurses), working with issues surrounding genomic research in Denmark, including the Faroe Islands, and persons with schizophrenia. The informants in the focus group interviews comprised persons with ADHD, parents to children with ADHD, clinical geneticists, and healthy controls (blood donors from the Danish Blood Donor Study (DBDS)). The persons with schizophrenia and ADHD were all diagnosed by psychiatrists and were stable in daily life. All blood donors who participated in the interview (and in the survey) are part of DBDS and have all given a broad informed consent to participate in a biobank for research and are potential healthy controls in genomic research. To be included in DBDS, the individual is not subjected to any medical treatment [33, 34]. Participants in the interview were all voluntary and informants were recruited through direct email contact, paper flyers at an activity center for mentally ill, invitations posted in an ADHD Facebook group, an ADHD clinic, a psychiatrist working in private practice, Copenhagen Hospital Biobank, and a blood donor Facebook group.

### The data analysis

All interviews were audio-recorded, transcribed verbatim, coded, and analyzed using the software package NVivo 11 [35]. NVivo helped to organize and manage the data. We were based on Grounded Theory as a method to analyze the data because this analytical strategy suggests a systematic analysis and enabled us to explore and identify attitudes towards inclusion of children in genomic research [36]. All transcripts were analyzed after a three-step process: open coding, axial coding, and selective coding [36]. We examined the emerging core concepts overall and how they differed across informants. We used some of the core concepts to develop items for the survey. For this paper, we selected relevant and illustrative quotes regarding inclusion of children in genome research and return of results. All quotes were translated from Danish into English by a professional translator.

## The cross-sectional survey

After the interviews, we conducted a cross-sectional web-based survey to explore specific attitudes towards the use of genomic research in Denmark. The survey was a translated and modified version of an English web-based survey developed at the Wellcome Trust Sanger Institute in Cambridge, UK [3, 37–40]. Details of the development of both the English and Danish survey versions have been presented elsewhere [39, 41]. On basis of the qualitative interviews, we modified the Danish version of the survey to also include items on informed consent, inclusion of children, and personal and familial experience with mental disorders. Items on children in genomic research were included because it was a key topic in the interviews and because the category of “age” came up in all the interviews. The survey also included self-reported sociodemographic information. Ten explanatory video films with subtitles and voice-over were used to illustrate the ethical issues raised in the survey (https://svaros.dk/holdning). The data collection began in August 2014 and ended April 2015. An average survey took approximately 23 min to complete.

### The stakeholders

Potential stakeholders in psychiatric genomic research were recruited: (i) 241 persons with mental disorders (who are potential cases in genomic research), (ii) 671 relatives to individuals with mental disorders (who are potential controls in genomic research), (iii) 1623 blood donors from DBDS (who serve as healthy controls in genomic research) [33, 34], (iv) 28 clinical geneticists (who analyze, return, and explain genomic data to patients and relatives in their clinical work and who must validate sequencing findings obtained in research), and (v) 74 psychiatrists (who diagnose and treat people with mental disorders) (*N* = 2637). We recruited the stakeholders through email, paper flyers at psychiatric hospitals, invitations posted in an ADHD Facebook group, and links at the homepages of the Danish Psychiatric Association, the Danish Society of Medical Genetics, and user groups of psychiatric patients and their relatives.

### Statistical analysis

We expected parents to hold a different attitude from non-parents because of their parenthood and interest in the health of their child. For example, parents might be motivated to participate in genomic research with the hope of receiving crucial information about the health of their child. Therefore, we decided to divide the stakeholders into parents (persons with children under age 26 years living at home) or non-parents (persons without children).

**Fig. 1:**
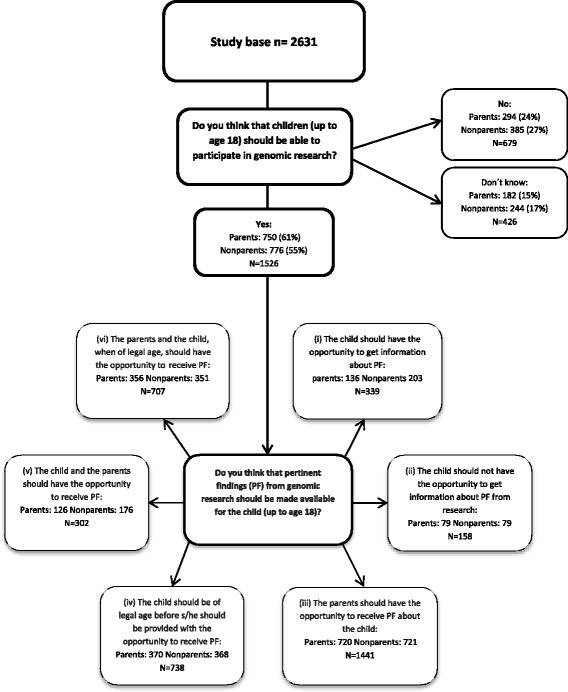
Flowchart of attitudes towards the return of pertinent findings to children or parents distributed on parents and non-parents, *N* and %

**Fig. 2:**
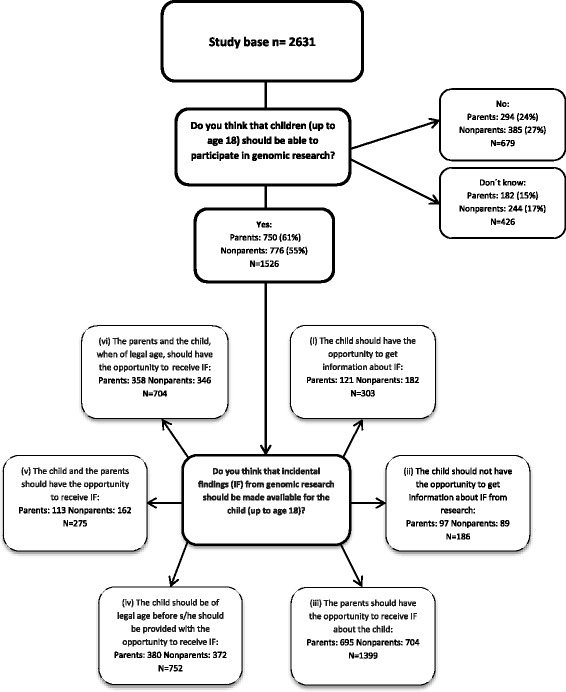
Flowchart of attitudes towards the return of incidental findings to children or parents distributed on parents and non-parents, *N* and %

Descriptive statistics were used to characterize the study sample. Unadjusted associations between items and stakeholders were estimated using *χ*^2^ tests. Multivariable logistic regression with 95% confidence interval (CI) and a *p* value of 0.05 was used to estimate the association between item and stakeholder adjusted for parenthood, gender, age, educational level, marital status, and stakeholder group. “Don’t know” answers were omitted from the regression model but are a part of the analysis regarding the questions towards the return of pertinent and incidental finding in children (Figs. 1 and 2). The analysis of the data was carried out using SAS® 9.4 [42].

The two questions regarding return of pertinent and incidental findings from research could be answered in four different ways, and respondents were allowed to choose more than one answer. We analyzed all answers and identified six combinations for pertinent findings and the same six combinations for incidental findings: (i) The child should have the opportunity to get information about pertinent/incidental findings, (ii) The child should not have the opportunity to get information about pertinent/incidental findings from research, (iii) The parents should have the opportunity to receive pertinent findings about the child if they would like to receive such information, (iv) The child should be of legal age before getting the opportunity to receive pertinent/incidental findings, (v) The child and the parents should have the opportunity to receive pertinent/incidental findings, (vi) The parents and the child, when of legal age (18 years), should have the opportunity to receive pertinent/incidental findings (Figs. 1 and 2).

## Results

### The qualitative interviews

#### Sample characteristics

A total of 10 semi-structured qualitative interviews were conducted in this study: four focus-group interviews, one group interview, and five individual interviews. The informants consisted of 12 males and 17 females (Table 1).

**Table 1:** Overview of sample characteristics in the qualitative interviews

Interview	Informants	Supplementary information
Focus-group interview	Persons with ADHD (*n* = 5)	Male 4; female 1, Denmark
Focus-group interview	Parents to children with ADHD (*n* = 6)	Male 1; female 5, Denmark
Focus-group interview	Danish blood donors from DBDS (*n* = 5)	Male 3; female 2, Denmark
Focus-group interview	Clinical geneticists (*n* = 6)	Male 1; female 5, Denmark
Group interview	Experts (*n* = 2)	Females, Faroe Islands
Individual interview	Expert	Female, Faroe Islands
Individual interview	Expert	Male, Denmark
Individual interview	Person with schizophrenia	Female, Denmark
Individual interview	Person with schizophrenia	Male, Denmark
Individual interview	Person with schizophrenia	Male, Denmark

The table presents type of interview and background of informants, their gender, and country of origin

#### Attitudes towards using children as genomic research subjects

The attitudes towards children as genomic research subjects varied. Experts, persons with ADHD, and parents of children with ADHD generally had a positive view, whereas clinical geneticists, persons with schizophrenia, and blood donors tended to have more ambiguous views. As a person with schizophrenia expressed: “Children should not be part of a research project, really. That makes a mess.”

The age of the child played a major role in the talks and discussions about children as research subjects. The overall attitudes were that parents should make the decision on whether their child should be included or not, that parents should give informed consent on behalf of their child, and that the child should be involved in the decisions depending on the age of the child: the older the child, the more the child should be involved in the decision-making.

The willingness to enroll children in genomic research was often associated with the dilemma of receiving the individual results:

Is it fair to deny the child the right to receive this knowledge [about genetic findings] from a research project – to say yes or no regarding information about himself or herself? […] I don’t think that we should cancel the research, but I think that the problem occurs when you start to involve the families in the return of individual results. (Clinical geneticist)

During the interview with the clinical geneticists, the discussion addressed the use of PKU samples in research and two informants discussed use of their own children’s archived neonatal PKU samples:

Clinical geneticist A, “My children, they should no longer be registered in the Danish Neonatal Screening Biobank. I will make the call next week.”

Clinical geneticist B, “What then if they become ill, and you could have known it?”

Clinical geneticist A, “Yes. I know that.”

Clinical geneticist B concludes, “But it is exactly post-rationalization that is difficult. I mean, beforehand, we may say that we don’t want to know, but what then if we suddenly have a sick child; and we could have done something?”

The clinical geneticists discussed the return of individual research results in relation to a child’s right to make an autonomous decision, but the discussion also touched on the possibility of using the research results as clinical information with potential health benefits for their child.

Several informants agreed that there is an emotional burden linked to the recipient’s age at the time when information about such findings is received and that returning the findings too early in the child’s life “could ruin the person’s life.” (blood donor)

There is great difference between a man of 51, such as myself, with certain life experience – both good and bad – and a young person with certain ideas about how hers life should be and who would be terribly disturbed by knowing of an 80 percent risk of getting, for example, breast cancer. So you have to consider when in life you should [return knowledge about findings], I think that is what I believe. (Other blood donor)

Some of the informants also expressed that there is an emotional burden associated with the return of findings to parents:

Well, I would say that neither I nor the children should know anything before they have turned 18. I really don’t think that the children can handle knowledge about it at their age and I… I should obviously not have that knowledge about my children before they are 18. Because I cannot handle such knowledge either. (Mother of a child with ADHD)

Nevertheless, there was no consensus among the informants as to whether the parents or the child should receive the findings:

There is no doubt that I love him, my son. I love him more than anything, but I would still like to have that knowledge because it is a hard life. (Father of a child with ADHD)

The two parents of children with ADHD have different attitudes to the return of findings from research. The mother states that she should not receive the findings until her child is of legal age, whereas the father focuses on the opportunity to receive information that could help his son get a better life.

In short, the attitudes towards children as genomic research subjects varied among informants. There was no consensus whether children should be genomic research subjects and whether the child or the parents should receive the genomic findings. The attitudes of involving children in genomic research focused on the child’s right to take an autonomic choice when of legal age, the emotional burden of knowing of both children and parents, and the possibility of receiving clinical information that could benefit the future health of the child.

### The survey

#### Sample characteristics

A total of 1227 parents and 1406 non-parents completed the survey. Their characteristics are presented in Table 2. There was an almost equal distribution between males and females. Parents were significantly older than non-parents. The mean age was 45 years (standard deviation (SD) 8) for parents and 48 years (SD 15) for non-parents. A higher proportion of parents than non-parents had a long level of education and was married/cohabiting. The majority of persons with mental disorders were diagnosed with depression, anxiety, or obsessive compulsive disorder by a doctor. The majority of relatives was first or second degree relatives (data not shown). No statistically significant differences were found for gender and stakeholder group membership between parents and non-parents (Table 2). Thus, parents were older, better educated (due to their age), and—not surprisingly—married.

**Table 2:** Socioeconomic characteristics of survey respondents

	Parents (*n* = 1227)	Non-parents (*n* = 1406)	*p* value**
	% (n*)	% (n*)	
Sex			0.37
Female	52 (634)	53 (751)	
Male	48 (591)	47 (653)	
Age groups, years			*< 0.0001*
20–40	28 (347)	36 (504)	
41–60	70 (852)	37 (526)	
61–76	2 (25)	27 (373)	
Age, mean (SD)	45 (8)	48 (15)	
Level of high education***			*< 0.0001*
None higher education	2 (17)	3 (34)	
Short higher(< 3 years)	28 (348)	29 (409)	
Medium higher (3–4 years)	29 (356)	32 (449)	
Long higher (> 4 years)	38 (470)	31 (439)	
Other education	3 (35)	5 (74)	
Marital status			*< 0.0001*
Married/cohabiting	84 (1032)	52 (727)	
Partnership	6 (70)	17 (241)	
Single	10 (122)	31 (437)	
Stakeholder groups			0.23
Persons with mental disorders	8 (103)	10 (138)	
Relatives to persons with mental disorders	26 (322)	25 (348)	
Blood donors	61 (749)	62 (871)	
Clinical geneticists	2 (18)	1 (10)	
Psychiatrists	3 (35)	2 (39)	

Socio-economic characteristics of the sample; specified in percentage and number of participants divided by parents, non-parents, and total. Values that were significant at *p* < 0.05 are set in italics

*n varies because of missing data

**χ^2^

***Children must receive 10 years of compulsory education in Denmark

#### Attitudes towards children as research subjects

Table 3 shows the respondents’ attitudes towards children as research subjects in genomic research and the association with parenthood, age, educational level, marital status, and stakeholder group.

**Table 3:** Attitudes towards children as research subjects in genomic research

Do you think that children (up to age 18) should be able to participate in genomic research?				
	*N*	Yes % (*n*)	OR _adj_ (95% CI)*	*p* value
Parenthood	2205			
Non-parent		67 (776)	1.00	
Parent		72 (750)	1.19 (0.96–1.48)	0.12
Sex	2204			
Female		63 (714)	1.00	
Male		76 (813)	1.77 (1.46–2.14)	*< 0.0001*
Age groups, years	2202			
41–60		70 (806)	1.00	
20–40		69 (501)	0.95 (0.76–1.19)	0.67
61–76		68 (218)	0.92 (0.68–1.24)	0.58
Level of high education	2206			
Long higher (> 4 years)		73 (562)	1.00	
None higher education		60 (25)	0.54 (0.29–1.06)	0.07
Short higher (< 3 years)		67 (424)	0.75 (0.59–0.96)	*0.03*
Medium higher (3–4 years)		69 (456)	0.87 (0.68–1.11)	0.26
Other education		61 (60)	0.59 (0.38–0.93)	*0.02*
Marital status	2204			
Married/cohabiting		70 (1034)	1.00	
Partnership		70 (186)	1.09 (0.79–1.50)	0.62
Single		65 (305)	0.95 (0.75–1.22)	0.69
Stakeholder group	2207			
Blood donors		70 (956)	1.00	
Persons with mental disorder		66 (130)	1.00 (0.73–1.39)	0.99
Relatives		70 (386)	1.05 (0.84–1.31)	0.69
Clinical geneticists		67 (18)	0.88 (0.40–2.12)	0.77
Psychiatrists		69 (38)	0.84 (047–1.59)	0.59

Multivariable logistic regression was used to assess the attitudes towards children as research subjects in genomic research and the association with parenthood, stakeholder group, gender, age, educational level, and marital status, with 95% CI and *p* value of < 0.05. Values that were significant at *p* < 0.05 are set in italics

*Adjusted for parenthood, stakeholder group, gender, age, educational level, and marital status

Significantly more males (76%) than females (63%) and significantly less persons with short higher education (67%) and other education (61%) than persons with long higher education responded that children should be able to participate in genomic research.

A consistent finding was that parents were more positive towards this statement than non-parents. Persons in the young age group (20–40 years), the older age group (61–76 years), single persons, persons with mental disorders, psychiatrists, and clinical geneticists were more negative towards the statement than persons in the mid age group (41–60 years), persons in a partnership, and blood donors (Table 3).

#### Attitudes towards the return of pertinent and incidental findings in children

As shown in Table 3, a majority of parents and non-parents responded that children should be able to participate in genomic research. However, 24% parents and 27% non-parents responded that children should not be able to participate in genomic research, whereas 15% parents and 17% non-parents did not know (Figs. 1 and 2).

The parents and non-parents who responded in the survey that children should be able to participate in genomic research (*N* = 1526) were asked about their opinion on the return of pertinent and incidental findings (Figs. 1 and 2). Firstly, both for pertinent (*N* = 1441) and incidental findings (*N* = 1399), parents and non-parents agreed that the parents should have the opportunity to receive both types of findings. Secondly, the child should be of legal age before s/he should be provided with the opportunity to receive pertinent (*N* = 738) and incidental findings (*N* = 752).

## Discussion

### Main findings

Two main discussion points emerged from the study. These will be discussed below.

### Children as genomic research subjects

In a hypothetical scenario, participants are overall likely to hold positive views on the question whether a child should be able to participate in genomic research. Our findings are similar to Fernandez et al. [26], who found that the majority of participants reported that children should be able to take part in genomic research, whether the condition under study began in childhood or not and independent of the existence of effective treatment. We expected that parents in the survey would hold a more positive view and that non-parents would be more indifferent, but we found only slightly higher agreement among parents than among non-parents.

There was a significant higher agreement towards involving children in genomic research among males than females indicating that males have less protective or more chance-taking behaviors than females. Having short higher education and other education was significantly associated with less agreement toward the statement than persons with long higher education. Individuals with low level of education may have greater difficulties in understanding the consequences of genomic data and thus more worries of letting children participate in genomic research.

#### Return of results: the child’s right to know

Participants expressed an attitude and also an expectation towards receiving research findings concerning children. In the interviews, there was no consensus whether the child or the parents should receive the findings. However, the return of findings were discussed in relation to the child’s age, the child’s right to an autonomous choice, the possibility of the emotional burden of knowing, and the possibility of receiving important clinical information. The majority in the survey agreed that both pertinent and incidental findings should be returned to the parents and to the child when of legal age. As parents might be more interested than non-parents in receiving clinical information, we expected that parents would take a more positive approach than non-parents. Nevertheless, we found that parents and non-parents had very similar attitudes. Thus, having children does not seem to affect stakeholder’s attitudes towards return of findings in children.

Furthermore, there were no differences in participant’s attitudes towards the return of pertinent or incidental findings, and they are interested in receiving both types of findings regarding the child. Our results illustrate that there are an interest in receiving information and that the child’s right to an autonomous choice and the parent’s interest to be informed are both important for the participants. The results in the survey study are consistent with those of Kleiderman et al. [28], who found that parents of children affected by a wide range of rare diseases expressed an interest in receiving their child’s research results. However, our results further illustrate that the guideline on genomic research from The Danish National Committee on Health Research Ethics [25] is more restrictive than participants’ attitudes toward the inclusion of children in genomic research. The participants accept inclusion of children in research without having direct health benefit for the child, including studies of the genetics of a disorder. Additionally, they are more positive towards the return of findings than the guideline.

Although the child does not have full autonomy when joining a genomic research project, the child will have autonomy in the future as an adult. As genomic research may provide clinical findings about both child-onset and adult-onset genetic diseases, it is important to consider the child’s future and right to know and not to know. Respecting the right to an open future means that the child can make his or her own autonomous decisions when reaching adulthood [10, 14, 27, 43, 44]. Feinberg [45] holds that children have a right, while they still are children, to remain ignorant of disease predisposition until they reach adulthood, presumably capable of making a well-informed decision. While the child is still a child, these “future interests” include interests that the child will in fact come to have in the future, including future interests that might never happen [45]. The right to an open future protects the child against having important life choices determined by parents (and others) before having the ability to make them for themselves. The parents can disrupt the child’s right to an open future if they act in a way that will cut off possibilities for the child in adulthood. For example, if the parents receive findings regarding breast cancer because of mutations in *BRCA1* or *BRCA2* genes, it can conflict with the child’s right to an open future if knowledge is disclosed that the child would have wanted to live without as an adult. Nevertheless, withholding discoverable information from the child could also close a child’s future if the child were interested in receiving the information as a child. The legal regulations in Denmark are unclear whether the return of genomic results encompasses adult-onset disorders and whether the results should be returned to the child or to the parents. Thus, it is uncertain whether parents have a right to be informed about all their child’s genomic research findings, including adult-onset genetic diseases. The Clinical Sequencing Exploratory Research (CSER) Consortium and the Electronic Medical Records and Genomics (eMERGE) Network aim to develop practical strategies for addressing questions concerning the return of results in genomic research [44]. For example, CSER and eMERGE address whether adult-onset findings should be offered for pediatric research participants. They conclude that, during the consent process, the parents should be offered the choice of whether or not to have the adult-onset actionable incidental findings returned along with counseling [44]. In the Danish clinical setting, children are not being offered genetic testing for adult-onset conditions. However, the Danish legislation in this area is unclear concerning the research setting. The results of this study suggest that it is important to incorporate the child’s right to an open future and to address the return of information of adult-onset genetic diseases in the (legal) discussion of children as research subjects.

### Strengths and limitations

It is a strength of the study design that we used both interview and survey data because the two methods contribute different insights to the topic. The interviews provided an in-depth perspective concerning the attitudes and views of the informants, and the survey data provided a broader and more general attitudinal perspective. It is also a strength that the first part of our data collection was used to inform the second part as the qualitative interviews inspired us to include additional items in the questionnaire survey to focus its scope. In the survey, we were measuring genomic research broadly and not specific at psychiatric genomic research. However, no larger studies have investigated the attitudes of patients, relatives, and health professionals in psychiatry and genetics towards inclusion of children in genomic research. The results illustrate that persons with mental disorder and relatives do not differ in attitudes from blood donors or clinical geneticists. This study thus contributes with new original knowledge in relation to stakeholders in psychiatry in Denmark. Each mode of the data collection provided preferential access to certain parts of the population. It is a strength that we used web-based survey since this method is known to recruit hard-to-reach groups [46, 47]. At the same time, using a web-based survey requires the participants to be familiar and have access to smartphones, tables, laptops, or computers, and this may have excluded potential participants without these skills or without access to such technical equipment or to the internet. Therefore, it is likely that the included stakeholders comprise a more homogeneous group, more positive of genomic research than potential participants who did not participate.

The study had some further limitations. Firstly, the survey and interviews had the inherent weaknesses of measuring hypothetical scenarios as the participants did not have direct experience with genomic research. “Real” requests to use children in genomic research could produce different results. Responses to hypothetical scenarios often anticipate behaviors and future intentions rather than actual behavior [48]. Therefore, studies are needed to test actual behavior in a real-life situation. However, we still think hypothetical scenario methodology is an important tool for predicting interest in research and to understand attitudes. Secondly, participants were included on a voluntary basis, and we cannot assess the effects of non-response. Participants may be more favorably inclined towards genomic research than the general population since the participants must be willing to take part in this study and thereby have an influence on the generalizability of the study results. The survey might have been too difficult or too long to maintain the engagement of the participants. This potential selection bias could mean that the results do not represent all possible stakeholders in genomic research in Denmark. Thirdly, persons with schizophrenia are difficult to recruit into trials [49]. In this study, it was difficult to recruit persons with schizophrenia to a focus-group interview. As a result, we decided to conduct individual interviews in this group of informants. A focus-group interview allows for interaction between the informants and may have given some more knowledge about the mechanisms behind certain attitudes expressed by the informants. Furthermore, it is possible that other core concepts would have emerged including persons with different mental disorders and psychiatrists because it is not certain that the focus-group interview went to saturation with the included interviews. Fourthly, we used a broad age category of children in the survey. As the age of the child could influence the participant’s attitudes, it is important to divide the children into several age categories in future studies. Fifthly, the participants were not specifically recruited because of their status as parents or non-parents. Instead, in consideration of the overall aim, we focused on recruiting a wide range of stakeholders in psychiatric genomic research: persons with mental disorders, relatives, blood donors, psychiatrists, and clinical geneticists. Finally, it could have been interesting to study the participants’ attitudes towards the return of pertinent and incidental findings in children subdivided into childhood-onset and adult-onset diseases.

## Conclusion

Participants generally reported positive views on the inclusion of children in genomic research. Additionally, our results illustrate that both the child’s right to autonomy and the parent’s interest in receiving information are important and valuable factors for the participants. They hold more positive and comprehensive views than the current Danish Guidelines on the inclusion of children and return of findings. However, having children does not affect the participant’s attitudes towards the inclusion of children as research subjects in genomic research.

Genomic research on children raises complex ethical issues and it is important to consider the view of potential stakeholders who rarely get a voice. Despite the Danish Guidelines, the issue of when to include children as research subjects and how to deliver research findings is still unclear and subject to legal challenges. We hope that sharing their attitudes will help the mobilization of knowledge on children as genomic research subjects, the return of findings regarding adult-onset genetic diseases, and the children’s right to an open future. Similarly, we think it is important to address these issues in the legal discussion of children as research subjects.
